# Turning Strategy into Action – Using the ECHO Model to Empower the Australian Workforce to Integrate Care

**DOI:** 10.5334/ijic.7036

**Published:** 2023-05-17

**Authors:** Perrin Moss, Phil Nixon, Sarah Baggio, Dana Newcomb

**Affiliations:** 1 Bachelor of Business, Bachelor of Creative Industries, ECHO Program Manager, Children’s Health Queensland, and Doctor of Philosophy candidate, The University of Queensland, PO Box 3474 South Brisbane 4101, Queensland, Australia; 2 Bachelor of Physiotherapy, Master of Development Practice, Graduate Certificate in Clinical Education, ECHO Network Coordinator, Children’s Health Queensland, PO Box 3474 South Brisbane 4101, Queensland, Australia; 3 Bachelor of Health Sciences (Physiotherapy), Honours Bachelor of Kinesiology (minor Gerontology), ECHO Network Coordinator, Children’s Health Queensland, PO Box 3474 South Brisbane 4101, Queensland, Australia; 4 Bachelor of Medicine, Bachelor of Surgery, Diploma of Child Health, Fellow of the Royal Australian College of General Practitioners, Medical Director Integrated Care, Children’s Health Queensland, and Senior Lecturer, Primary Care Clinical Unit, The University of Queensland, PO Box 3474 South Brisbane 4101, Queensland, Australia

**Keywords:** integrated care, Project ECHO, communities of practice, paediatrics, interprofessional education, workforce

## Abstract

**Introduction:**

Children’s Health Queensland (CHQ) established a telementoring hub in Queensland, using the Project ECHO® model, to pilot and scale a range of virtual communities of practice (CoP) to empower the Australian workforce to integrate care.

**Description:**

The establishment of the first Project ECHO hub in Queensland facilitated the implementation of a variety of child and youth health CoP that strategically aligned to the organisation’s approach to integrate care through workforce development. Subsequently, other organisations nationally have also been trained to implement and replicate the ECHO model to effect more integrated care through CoPs in other priority areas.

**Discussion:**

Findings from a database audit and desktop analysis of project documentation highlighted that using the ECHO model was effective in establishing co-designed and interprofessional CoP to support a cross-sector workforce to deliver more integrated care.

**Conclusion:**

CHQ’s use of Project ECHO highlights an intentional approach to establishing virtual CoP to build workforce capability to integrate care. The approach explored in this paper highlights the value of workforce collaboration amongst non-traditional partners to foster more integrated care.

## Introduction

Achieving integrated and high-quality healthcare for children and young people in the geographically dispersed population of Australia is challenging. Fragmented healthcare negatively impacts on health outcomes for the child, associated with increased caregiver burden, increased emergency department presentations, unplanned hospital admissions, and longer hospital stays [1,2,3,4]. Health policy and executive decision-makers are consistently challenged by the evolving demand for paediatric healthcare services outstripping supply [5]. This has been exacerbated by a growing paediatric population of children with medical complexity, representing high healthcare costs and resource utilisation [6]. More broadly, an ageing population and rising prevalence of chronic disease across Australia has led to system leaders driving reform agendas [7]. Despite the push to reform, legacy funding models have continued to struggle to incentivise the new ways cross-sector workforces need to collaborate to deliver integrated care, particularly across the primary-secondary healthcare continuum [8,9]. In Australia, similar to other developed countries, new funding investment to encourage more integrated care has also typically focused on short-term, intensive initiatives with narrow scopes which have tended to limit longer-term change [7,10].

The delivery of paediatric healthcare, as in adult healthcare, is becoming increasingly specialised and has led to fragmentation across the system. In the case of children and young people living with co-morbid and/or complex conditions, they often navigate multiple service providers, organisations, and sectors to have their health needs met. This service and policy reform landscape is further polarised by a reliance on innovators to create and harness new technologies to share best practices to integrate care amongst growing resource scarcity [11]. The case for enabling more integrated approaches to care is no more relevant than in Queensland, Australia. For context Queensland is the second largest state in Australia, with an area of over 1.7 million square kilometres [12]. By comparison, Queensland amounts to nearly five times the size of Japan, seven times the size of Great Britain and two and a half times the size of the state of Texas in the United States of America [12]. More than half of the population is geographically dispersed outside of the greater metropolitan area of Brisbane, across regional, rural, and remote communities. This distribution compounds the service delivery challenges of integrating care for children, young people, and their families. It is commonplace for many families to travel significant distances via car, train, or plane to access subspecialist paediatric care.

Children’s Health Queensland Hospital and Health Service (CHQ), based in the capital of Brisbane, is one of sixteen hospital and health service organisations in Queensland, and has a state-wide remit for providing specialist paediatric care [13]. In alignment with the Quintuple Aim and the World Health Organisation’s European Framework for Action on Integrated Health Services Delivery, CHQ developed an Integrated Care Strategy in 2018 [14,15,16,17]. The strategy included a practical tool kit to support the organisation to conceptualise and approach integrated care with a global definition and strong focus on workforce planning, education and training [18]. The strategy acknowledged that the health and wellbeing of children and young people was not exclusively dependent on their access to timely, safe, and effective medical care. Non-medical service providers such as those operating within the child safety, education, disability, and youth justice systems, as well as partnership with families were identified as being fundamentally important to effect outcomes associated with improved integration of care [18]. The design of the strategy created an organisational mandate to work at a systems level in collaboration with inter-agency and interprofessional partners and consumers to improve and enable the integration of services across geography, sector, and professional discipline.

CHQ defined integrated care for children and young people as:

“*The provision of care in the broadest sense – physical, psychological and social – which is oriented around the needs of children, young people and families, and designed and delivered in partnership with them. In an integrated system, these needs are met through the coordinated and collaborative working of all providers, irrespective of sectorial, organisation or geographic boundaries*” [18]

Integrated and child-centred models of care have been shown to have the potential for reducing fragmentation, improving effectiveness and sustainability of services and agency partnerships, while improving the care experience and outcomes for children, young people, and their families [7,8,19,20]. Through the development of the Integrated Care Strategy, CHQ identified a virtual model of capacity building and interprofessional education or telementoring, called Project ECHO®. CHQ adopted the ECHO model™ to enable the organisation to integrate the way care could be provided and enhanced at scale. The aim of this integrated care case is to highlight CHQ’s use of the ECHO model to enable more integrated care through the interprofessional learning, collaboration and practice supported by virtual communities of practice (CoPs). This case illustrates CHQ’s use of ECHO as one solution to alleviate the pressures and respond to the evolving needs experienced by the workforce.

## Ethical approval

This study was approved by the Children’s Health Queensland Human Research Ethics Committee under reference numbers: HREC/17/QRCH/67 and LNR/18/QCHQ/44762.

## Description of the care practice

### What is Project ECHO?

Project ECHO (Extension for Community Health Outcomes) is a synchronous virtual hub-and-spoke knowledge sharing model, connecting frontline professionals from any location with interprofessional panels of content experts, to build capacity at scale in real time [21]. Organisations that are licensed to use the ECHO model can launch ECHO Networks to connect and support the sharing of knowledge and best practices, often with particular focus on supporting frontline providers working in underserved or disadvantaged communities [22,23,24].

### How does ECHO work?

ECHO networks function with a hub-and-spoke model consisting of a panel of relevant content experts with a facilitator based at a hub organisation to guide the discussion and foster interactivity with the spoke participants who can be located anywhere [25]. ECHO sessions typically include a brief presentation by one of the panellists, on a topic identified as a priority learning area by the group. In addition, spoke participants who volunteer to join networks can present de-identified cases from their local practice, to seek advice from their network peers and panel members. Problem-based case scenario discussions provide an opportunity for all participants and panel members to contribute their knowledge and experiences to support their network peers. The scenarios elicit valuable information from the perspectives of unique disciplines, services, and sectors, building on knowledge of systems and sectors and presenting new opportunities for coordination and collaboration between providers. A summary of the co-designed recommendations suggested in response to each case are circulated to all participants, as a record of the key best-practices and opportunities for care integration to be considered for the presented case as well as similar case scenarios in other settings. The development of the ECHO model as a continuous learning solution has been underpinned and informed by the key principles of Community of Practice Theory, Social Cognitive Theory, and Situated Learning Theory [26,27].

Through this methodology, organisations use ECHO to create virtual CoP that function as a continuous learning system that reinforces best practices and integration at scale [28,29]. By harnessing scarce content expertise and making it available at scale via freely accessible videoconferencing technology, at no cost to participants, ECHO moves knowledge rather than people. This also removes historical challenges of geographical and financial barriers to accessing support. The learner-centric approach inherent within the ECHO model’s methodology supports hub organisations to design and evolve their networks to meet the learning objectives of participants. This highlights how models such as ECHO can demonstrate their value to the current and future integrated care workforce by responding to their needs in an ongoing and targeted way.

### Establishment of the ECHO hub at CHQ

In 2016, a General Practice Liaison Officer from CHQ identified that the ECHO model would be a suitable platform to integrate care between interprofessional paediatric and primary care teams, especially General Practitioners (GPs) [18]. CHQ was the first Queensland organisation to implement the ECHO model and established an ECHO hub at CHQ’s Centre for Children’s Health Research in South Brisbane. This initiative was funded by the Queensland Department of Health’s Integrated Care Innovation Fund (ICIF) as a once-off grant to incentivise hospital and health service organisations to pilot innovative new models to integrate care [7,30]. CHQ harnessed the ECHO model to enhance GPs’ access to subspecialist advice and support at scale, by virtually connecting paediatric teams with interprofessional spoke participants that were treating and managing children and young people with complex health, disability, education, and psycho-social needs in communities across Queensland.

CHQ launched their first ECHO Network as a pilot in 2017 focussing on the health needs of children and young people with stable attention deficit hyperactivity disorder (ADHD) [9]. ADHD is common, affecting 5–7% of Australian children aged 4–18 years and representing 20% of all paediatric outpatient encounters nationally [31,32,33]. Historically, this has resulted in the ongoing management of children diagnosed with ADHD almost exclusively being provided by paediatricians [31,32,33,34,35,36,37]. Many children with ADHD are medically stable and could be safely managed by their GP. This represented an avoidable burden on outpatient resources, an unnecessary burden on families, and an increased risk of fragmented care.

Following significant improvement in primary care provider self-efficacy achieved in the ADHD ECHO Network pilot [9], CHQ launched multiple other ECHO Networks in quick succession across a variety of topic areas to reach frontline workforce participating from spoke participant learning sites at a national level. Qualitative data, including responses from single-session surveys, semi-structured interviews, subsequent learning needs assessments (LNAs), and in-session verbal feedback, provided reassuring evidence that the innovation improved GP and other spoke participants’ capacity and confidence to better manage children in local communities with advice and mentorship provided by the network peers and panel experts through the suite of CHQ ECHO Networks.

### The process of developing ECHO Networks

Project ECHO® is a learner-centric virtual hub-and-spoke model of education, based on the principle of “all teach and all learn” [25]. CHQ’s ECHO Networks have applied this principle to facilitate all participants, including hub panel members, being actively engaged in learning from one another’s expertise and insights, using a bi-directional exchange [38,39,40]. Spoke participants share their knowledge from local social and cultural reflections, paired with an understanding of realistic approaches to service provision within their specific communities [38,41]. The panel specialists offer complementary content expertise, and as a result the virtual CoP or ‘knowledge networks’ develop over time. As the ECHO Networks mature over time, they create a dynamic where each participant plays a role in co-producing and contributing to the knowledge pool which the collective can harness at scale [26,27]. This process also enables participants to develop new skills to manage complexities as they exist in their local context [26,27]. While much of the published literature about integrated care cites gaps and barriers that reinforce fragmented systems, CHQ’s ECHO Networks continue to serve as a universal demonstrator that historically disparate workforce can be effectively connected to integrate care [5,9,11,42].

CHQ ECHO Networks are designed to fulfil the three key elements of a CoP: (1) provide a clearly-defined *domain*, or common understanding of the boundaries of a shared learning agenda, (2) establish mutual respect and trust within the *community*, which allows for participants to share their own ideas and to expose gaps in their knowledge, and (3) invite contributions to the pool of knowledge or *practice* held and developed by the group, including the sharing of experiences, resources, professional contacts, and best practices [27]. The impact of CHQ’s ECHO Networks has depended on the commitment of spoke participants to any of the three key elements, and panel member investment in all three. The cumulative co-design and ongoing quality improvement activities of CHQ ECHO Networks evolve in line with each Network’s maturing understanding of CoP concepts.

CHQ’s ECHO Networks, consistent with other ECHO Networks globally, are co-designed in partnership with prospective spoke participants through an initial LNA, undertaken by the hub team to identify the topics that are of most relevance to address spoke participants’ occupational role challenges, local contexts, interests, and individual learning objectives [43,44,45]. The results of the LNA are then used to curate the ECHO Network’s initial didactic curriculum and identify suitable subject matter experts from within the organisation or external partners who can contribute their expertise as panellists. A continuous quality improvement cycle is then applied through periodic reviews to evolve the curricula to ensure it remains relevant to the learners’ needs.

The promotion of ECHO Networks and the participant membership is a key element of implementation planning that requires consideration and preparation to ensure the objectives of integrating care can be achieved. While the ECHO model has been in use since 2003, it remains a relatively new concept for most individuals, with the ‘elevator pitch’ being nuanced depending on the context of the ECHO hub organisation’s sector and organisational workforce priorities. As a result of these factors, the CHQ ECHO hub teams’ approach to promoting their networks required tailored messaging to attract prospective participants by piquing their interest with an invitation that directly benefits them as an individual within a particular workforce and serves as a call to action. To ensure the unique motivations of prospective spoke participants was catered to, an intuitive web-based enrolment portal was also developed to enhance responsiveness to the learning needs analyses for each ECHO Network. Similarly, to the promotional activities mentioned above, the onboarding resources and registration processes were also curated by the CHQ ECHO team to be intuitive and flow individuals to networks that responded to their learning objectives.

### Establishment of the ECHO Superhub at CHQ

With the ECHO model’s origins firmly based within the healthcare sector, it has since grown to over 1000+ licensed organisations globally across 68 countries [46]. Historically, the ECHO Institute, based at the University of New Mexico (UNM) in the United States of America, centrally managed the licensing and training of all new organisations implementing the ECHO model globally [42]. It was identified that this single training centre limited UNM’s capacity to meet demand. In response to this bottleneck, other established ECHO Hub organisations with successful track records were invited to serve as local training centres of excellence for diffusing the model globally [47,48]. The function of organisations designated as ECHO Superhubs is to facilitate licensing and provide formal partner launch training (formerly called Immersion) and mentoring to new organisations implementing the ECHO model in their own context [47,48]. CHQ’s implementation, expansion and ongoing operation as Australia’s largest ECHO Hub laid a foundation to be designated as the South Pacific’s first ECHO Superhub organisation in 2019 [47]. CHQ saw the Superhub designation as a strong catalyst to further enable its Integrated Care Strategy to actively support and mentor external teams from diverse sectors to adopt and implement the ECHO model as a universal enabler in the delivery of integrated care, innovation, and workforce development.

## Discussion

### CHQ ECHO Networks outcomes

**Figure 1:**
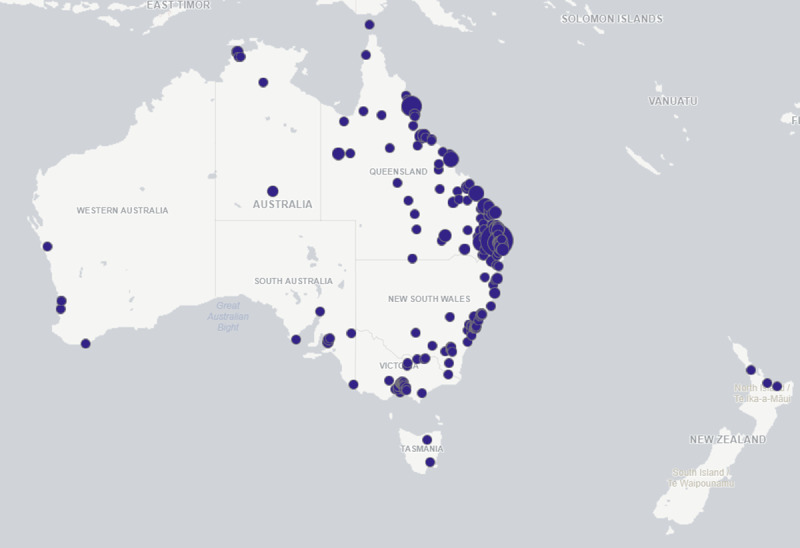
Geographic distribution of spoke participants joining CHQ ECHO Networks 2017- September 2022 (n = 2574).

Since the ADHD pilot, CHQ has expanded the use of ECHO to a total of 22 ECHO networks supporting spoke participants across professional disciplines, sectors, and geographies. The data illustrated in this integrated care case was collected via an audit of CHQ’s iECHO database and desktop analyses of project documentation, including single-session survey responses, subsequent LNAs and in-session verbal feedback. The iECHO database is a customised web-based partner relationship management tool that is used to collect, manage, and audit ECHO network performance. ECHO network data was collected and reviewed for the period 2017 to September 2022 inclusive, including participant demographics, attendance rates, case presentation themes and recommendations. Each network highlighted in Table 1 below has provided CHQ with the opportunity to experiment with the model’s application in a variety of contexts to support the identified workforce needs [11]. This has generated valuable learnings on the model’s utility across different target audiences, participant learning objectives, and measures of network success [42]. Since the early focus on capacity building for primary care providers, the value of interprofessional and cross-sector collaboration has been realised in the quality and sustainability of subsequent CHQ ECHO Networks. CHQ has harnessed the ECHO model to mentor and support over 2,574 individual members of the child and youth workforce across Australia and beyond. This impact is illustrated in Figure 1 below with participation figures listed in Table 1.

**Table 1:** Demonstrated examples of workforce diversity across CHQ’s ECHO Networks as of September 2022.


#	ECHO NETWORK AND NETWORK MISSION STATEMENT	YEAR OF LAUNCH AND STATUS	TARGET AUDIENCE: SECTOR DIVERSITY	TARGET AUDIENCE: PROFESSIONAL DISCIPLINE DIVERSITY	CUMULATIVE NUMBER OF UNIQUE SPOKE PARTICIPANTS; CASE PRESENTATIONS

1	**Attention Deficit Hyperactivity Disorder (ADHD)** *To support GPs to manage children with ADHD in collaboration with hospital teams and other primary care providers*.	2017 – 2019	Primary health, secondary health, education	GPs, psychologists, guidance officers	90; 81

2	**Paediatric Overweight and Obesity** *To prevent and manage childhood obesity in primary care with specialist support*.	2018 – 2019 (transitioned to partner ECHO hub for continuity)	Primary health, secondary health	GPs, dietitians	110; 41

3	**Clubfoot and other Foot Anomalies** *To build capabilities of physiotherapists and orthopaedic nurses managing clubfoot and other congenital foot anomalies that can be effectively managed outside tertiary centres*.	2018 – 2020	Secondary health, tertiary health	Physiotherapists, orthopaedic nurses	85; 53

4	**Early Years Development Program** *To train and mentor early childhood educators in administering universal child developmental screening and parental support*.	2018	Primary health, early childhood education	Early childhood educators	15; 5

5	**Refugee Kids** *To provide a forum for educators to access professional guidance and support to respond to the complex needs of refugee families impacted by trauma*.	2018 – 2019	Education, refugee health, non-government, secondary health	Principals, teachers, guidance officers, refugee advocates, school nurses	101; 32

6	**Kids & Teens Mental Health and Behaviou**r *To empower GPs and other professionals from health and education to manage behaviour and mental health within the context of the family and community environments*.	2018 – 2020	Primary health, secondary health, education	Medical, nursing, allied health, guidance officers	186; 69

7	**Children, Adolescents and Young Adults with Complex Pain** *To enable the delivery of developmentally sensitive, personalised care for every child, adolescent, and young adult with chronic pain*.	2018 – present	Primary health, secondary health, tertiary health, education, disability, non-government	Medical, nursing, allied health	187; 35

8	**Supporting Teams Caring for Type 1 Diabetes** *To improve and advocate for high quality and consistency in care for people living with T1D*.	2019, 2021 – present	Secondary health, tertiary health, education	Medical, nursing, allied health, school-based youth health nurses	90; 17

9	**Paediatric Palliative Care** *To improve the integration of high-quality care of children/young people with life-limiting conditions and their families. To grow an interprofessional community or practice, using a supportive environment, that enables us to gain and share knowledge and connect*.	2020 – present	Primary health, secondary health, tertiary health, education, human services, disability, non-government (hospice), education	Medical including GPs, nursing, allied health, education professionals	119; 17

10	**Autism Connect** *To assist educational professionals to support students with autism in Queensland schools, in response to the growing need for autism specific professional learning across the state*.	2020	Education	Principals, teachers, guidance officers	25; 6

11	**Supporting Kids and Families during and beyond COVID-19** *To provide a platform for health and education professionals to share information to support staff resiliency and student return to school*.	2020	Education, primary health, secondary health, child safety, non-government	Guidance officers, teachers, school nurses, allied health	144; 11

12	**Integrated Models of Care to support psychosocial needs during and beyond COVID-19** *To support the provision of healthcare to meet psychosocial needs during the COVID-19 pandemic*.	2020 – 2021	Primary health, secondary health, tertiary health, education	Social workers and other allied health professionals, medical, nursing	106; 4

13	**Adolescent Health and Wellbeing** *To support effective ways of working with adolescents and young adults to help build their capacity and build connections between professions and sectors to enable coordinated and collaborative care*.	2020 – present	Primary health, secondary health, tertiary health, education, child safety, non-government	Nurses including school-based nurses and nurse navigators, guidance officers and other education professionals, GPs, allied health, hospital medical teams, child safety professionals, youth workers	204; 23

14	**Good Grief This Hurts** *To build awareness around what to do when a child has experienced the death of a significant person in their life*.	2020	Secondary health, tertiary health, hospices, education	Anyone in the community who is caring for a child who has been impacted by death family members and friends, teachers, guidance counsellors, health care providers, private counsellors/psychologists, and community-based youth-focused organisations	253; 0

15	**Navigating Paediatric Disability** *To grow a statewide, interprofessional and cross sector community of practice. To connect and learn about cross sector, multi-agency services and supports available to better integrate care for children/young people who live with a disability*.	2020 – present	Primary health, secondary health, education, disability, child safety, non-government	Medical, nursing, allied health, child safety, disability, and education professionals.	183; 23

16	**Child Protection: Responding to Vulnerable Children and Families** *To build a village of professionals from health, child safety, education and our other partners to problem solve, share our successes and challenges in our work together responding to children who have been abused and/or neglected*.	2021 – present	Child safety, education, primary health, secondary health, tertiary health	Child safety officers and other child safety practitioners, child protection liaison officers, paediatricians, nursing, allied health, education professionals	185; 12

17	**Aboriginal and Torres Strait Islander Kids Health and Wellbeing** *To connect teams who care for Aboriginal and Torres Strait Islander kids to improve health and wellbeing*.	2021 – present	Aboriginal community controlled, primary health, secondary health, tertiary health, education, child safety	Medical, nursing, allied health practitioners, and Aboriginal and Torres Strait Islander Health Workers from Aboriginal and Torres Strait Islander Community-Controlled Health Services and mainstream services	119; 6

18	**Paediatric Gender Health Care** *To improve the knowledge and confidence of medical and mental health clinicians, education professionals and others to provide informed and respectful care to trans, gender diverse and non-binary youth*.	2021 – present	Primary health, secondary health, education	GPs, mental health professionals in health and education, child safety professionals	75; 10

19	**Paediatric Eating Disorders** *To improve access to support and develop a network around clinicians caring for children and young people living with eating disorders*.	2021 – present	Primary health, education	GPs, practice nurses, psychologists, school-based youth health nurses.	141; 3

20	**Paediatric Feeding** *To improve the knowledge and confidence of professionals working in paediatric feeding across Queensland*.	2021 – present	Secondary health, tertiary health	Speech pathologists, occupational therapists, psychologists, and dietitians	54; 16

21	**Replication and Beyond** *To support and develop knowledge sharing, capability and confidence of facilitators, panellists, and coordinators implementing the ECHO model within the Australian context*.	2019 – present	Primary health, secondary health, university, non-government, philanthropic, health promotion and prevention	Medical, nursing, allied health, project managers, administrators	85; 33

22	**Health Research Education** *To support knowledge sharing and advice to help grow and develop research and evaluation capacity*.	2022 – present	Secondary health, tertiary health, university, philanthropic	Medical, nursing, allied health, researchers/academics, and administrators	18; 3


It is important to note that while the number of unique spoke participants may appear small when compared to total workforce numbers, that participation in ECHO networks does not reflect a once-off encounter. Spoke participation in ECHO networks foster an ongoing opportunity for mentorship, peer support and collaborative learning over the longer term. Many of CHQ’s ongoing ECHO networks have retained spoke participation for greater than 12 months which suggests a value perception that is held by spoke participants.

Each ECHO Network listed in Table 1 has a Mission Statement that identified the unique focus of integration prioritised by the group. These statements were co-designed with spoke participants, and typically evolved as the CoP group developed a shared understanding of the challenges and opportunities of integrating care that was most pertinent to progressing enhanced integration outcomes collectively.

Table 1 highlights how CHQ’s use of the ECHO model has enabled the workforce to be connected, mentored, and supported by establishing virtual CoPs as one solution to address fragmentation across professional disciplines, organisations, sector systems and geographic silos. The findings can inform other replications of the ECHO model, particularly by teams in organisations locally and internationally to integrate care for children and adolescents.

### CHQ’s ECHO Superhub outcomes

CHQ’s credentialed Superhub designation in 2019 has enabled provision of local partner launch training for organisations in Australia and the South Pacific region seeking to adopt and implement the ECHO model to support the workforce to integrate care.

The CHQ team adapted and enhanced the standard partner launch training curriculum content to improve implementation success within the Australian context by incorporating a strong focus on integration of care and cross-sector workforce collaboration. Since this time, CHQ has trained several organisations across Australia to implement and replicate the ECHO model within other organisational and sectoral contexts. Table 2 highlights the geographic and sectoral diversity of Australian organisations that have adopted the ECHO model by training with the CHQ ECHO Superhub.

**Table 2:** Organisations trained by CHQ’s ECHO Superhub.


#	ORGANISATION	LOCATION	YEAR TRAINED	FOCUS AREA/S	YEAR LAUNCHED

1	Apunipima Cape York Health Council	Queensland	2019	Rheumatic Heart Disease, Maternal Child Health	2021

2	Health Consumers Queensland	Queensland	2019	Health Consumer training and support	2020

3	Royal Australian College of General Practitioners	Victoria	2019	Alcohol and Other Drugs	2020

4	Western Victoria Primary Health Network	Victoria	2019	Persistent Pain, Mental Health, COVID-19, Domestic and Family Violence, Movement Disorders	2020

5	Health and Wellbeing Queensland	Queensland	2020	Childhood Overweight and Obesity, Healthier Remote Food Stores, Tuckshop Menu Planning	2020

6	Townsville Hospital and Health Service	Queensland	2020	Persistent Pain	2020

7	The University of Melbourne	Victoria	2020	Optimising Chronic Disease Outcomes in Primary Care, Adolescent Mental Health, COVID-19 Vaccine Roll-out	2020

8	Thorne Harbour Health	Victoria	2020	Transgender Health	2020

9	Sydney North Health Network	New South Wales	2020	Pain Management, Palliative Care	2020

10	West Moreton Hospital and Health Service	Queensland	2020	Residential Aged Care, Specialist Mental Health and Intellectual Disability	2021

11	Darling Downs West Moreton Primary Health Network	Queensland	2020	Older Persons Health	2021

12	Clinical Excellence Queensland	Queensland	2020	Offender Health and Wellbeing	2020

13	Hunter New England and Central Coast Primary Health Network	New South Wales	2021	Comorbidity Support in Primary Care	2021

14	South Australian Postgraduate Medical Education Association	South Australia	2021	Alcohol and Other Drugs, Cardiology, Neurology, Chronic Pain, Gastroenterology	2021

15	Dokotela	New South Wales	2021	Psychiatry	ETA 2022

16	Western Australia Primary Health Alliance	Western Australia	2021	Alcohol and Other Drugs	2022

17	University of New South Wales	New South Wales	2021	Rare Diseases in Childhood, Ophthalmology	2022

18	Australasian Palliative Link International	Victoria	2021	Palliative Care	2022

19	Sunshine Coast Hospital and Health Service	Queensland	2021	Frail Elderly	ETA 2022

20	Northern Territory Primary Health Network	Northern Territory	2022	Disability	2022

21	Australian Centre for Prevention of Cervical Cancer	Victoria	2022	Cervical Cancer	2022

22	Metro South Hospital and Health Service	Queensland	2022	Frail Elderly, Residential Aged Care Facilities	2022

23	St Vincent’s Health Services	Victoria	2022	Adolescent Addiction	ETA 2023

24	GP Partners Australia	South Australia	2022	Palliative Care, Maternity	ETA 2023


In line with its Integrated Care Strategy, CHQ as an ECHO Superhub has trained many organisational teams across the primary, secondary, and tertiary healthcare sector to adopt the ECHO model. This has demonstrated the alignment and value proposition of the ECHO model to be harnessed by other organisations across sectors to intentionally increase the integration of care through workforce development. Through the provision of partner launch training, CHQ has been able to diffuse its cumulative expertise in implementing, sustaining, and expanding the use of the ECHO model to establish virtual CoPs to support new organisations across sectors and geographies.

While still being a paediatric healthcare organisation, CHQ’s role as an ECHO Superhub has been universal and agnostic, resulting in the steady growth of new hub organisations being established in a variety of other contexts, particularly throughout Australia. This has been achieved by providing organisations with access to localised partner launch training to replicate the ECHO model. These new adopters also shared CHQ’s organisational priority to build workforce capacity, knowledge sharing and service integration across the primary, secondary, tertiary, disability, health prevention and promotion sectors in their local contexts. Beyond the once-off partner launch training, CHQ has innovatively harnessed the virtual CoP methodology as the platform to efficiently provide ongoing mentorship, advice, and technical assistance for these organisational teams by establishing a dedicated ECHO Network in response to this workforce need via the Replication and Beyond ECHO Network (see Table 1). The diffusion achieved in Australia to date serves as encouragement that there is growing appetite amongst non-traditional, non-health sector partners to enhance integration across more sectors by adopting the ECHO model, and that the CHQ ECHO Superhub can support this diffusion into the future.

## Lessons Learned

1. The ECHO model can be successfully harnessed to establish virtual CoP to support the learning objectives of the workforce.
2. The case-based learning and interprofessional networking components of the ECHO Networks operated by CHQ have supported integrated care in action at scale across professional disciplines, sectors, and geography.
3. There is a growing interest in Australia for organisations to adopt the ECHO model to support the workforce to enhance how they collaborate to deliver integrated care.
4. Further research is warranted to investigate the wider implementation outcomes achieved by organisations adopting the ECHO model to integrate care in Australia; and
5. The ECHO Superhub plays an important role in supporting the successful diffusion of the ECHO model in different jurisdictions to scale the potential for more integrated care to be realised.

In addition to lessons learned, this Integrated Care Case also provides opportunity for future research pursuits to investigate:

1. Workforce retention outcomes.
2. Organisation/Systems change management outcomes.
3. Financial sustainability/cost neutrality of Project ECHO within public sector organisations.

## Conclusion

Globally, policymakers and executive decision-makers are contending with service demands exceeding supply, which at a systems level can be attributed largely to an ageing population and increased complexity. This requires innovative organisations to adopt new technologies and share best practices at scale. While CHQ’s organisational context and service delivery operates at the other end of the age continuum within the paradigm of the paediatric population, the organisation’s adoption of the ECHO model has demonstrated the dynamism and agility to implement non-traditional solutions to lead and facilitate more integrated workforce-led approaches to care by translating strategy into action.

This integrated care case highlights CHQ’s use of the ECHO model in workforce capacity building and inter-professional education, to enable more integrated care to be delivered at scale through virtual CoPs. As an organisation within the public healthcare system, CHQ adopted the ECHO model as an approach to service integration that was unique from other organisations at the time. Virtual CoPs were designed to build workforce capacity and share best practices at scale and remove traditional barriers of geography and professional/sectoral silos to accessing care.

CHQ identified the ECHO model as an enabler and exemplar of workforce development and integrated care that could be implemented and sustained in the Australian setting. Through cumulative experimentation and expansion, CHQ has gained extensive experience harnessing the ECHO model to achieve more integrated approaches to care by facilitating a growing number of virtual CoP where collaboration and knowledge sharing translates into practical outcomes across jurisdictions. In particular, problem-based case scenario discussions have provided a powerful tool for building each network’s knowledge of roles, services and systems, and a rich context to collaboratively discuss how knowledge translates into practice.

In CHQ’s role as an ECHO Superhub, these cumulative learnings have reinforced and underpinned the advantage and efficiency offered to new organisational teams completing partner launch training to implement the ECHO model in their own contexts from a reputable local expert. CHQ continues to reinforce the benefits of the model at a national level by using the ECHO model as the platform to also provide ongoing mentorship, advice and networking for implementation teams following partner launch training.

CHQ’s continued use of the ECHO model indicates an ongoing commitment to respond to the evolving needs of the integrated care workforce via virtual CoPs. In an organisational context, the ECHO Superhub function will facilitate other organisations to leverage the practice wisdom in implementing the model with success to realise more integrated care occurring across the continuum.
